# Supplementary material for: Social aloofness is associated with non-social explore-exploit decisions
Source: Commun Psychol. 2025 Jul 15;3:106. doi: 10.1038/s44271-025-00278-7 (PMC12263421; doi:10.1038/s44271-025-00278-7)
Supplement: Supplementary file 2 — Supplemental Information [file 44271_2025_278_MOESM2_ESM.pdf]

Supplemental Figures:

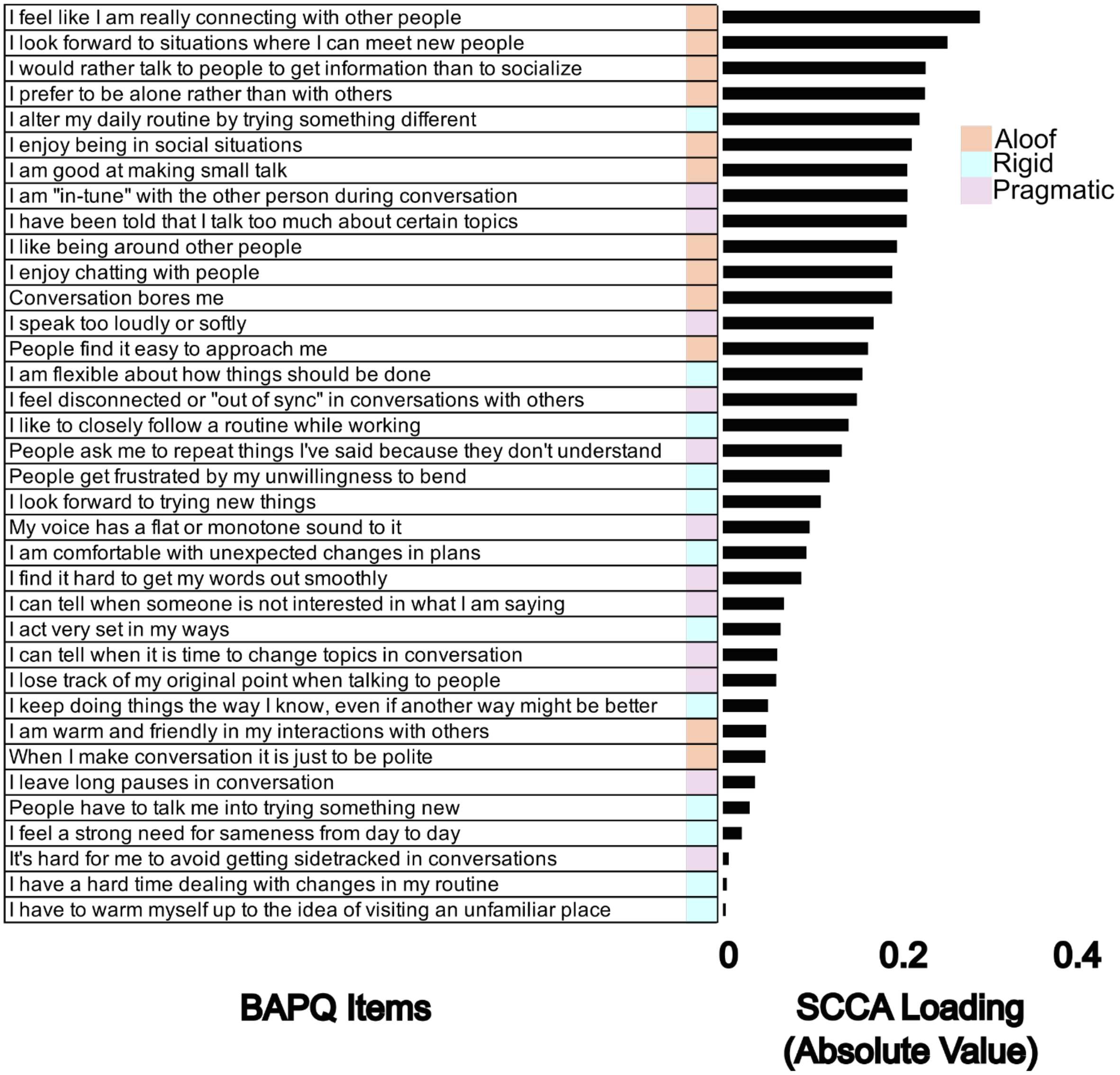

Figure S1: Sparse Canonical Correlation Analysis Reveals Behavioral Relevance of all 36 BAPQ Items

Left column contains the 36 questions within the BAPQ, with horizontal bars indicating the relative loading on a dataset consisting of nine behavioral indices: (1) p(explore), or percent of time spent in exploration, (2) exploration potential inferred from our hidden markov model, and the other six model-free indices included, (3) punishment sensitivity, (4) percentage of choices that were shifts, (5) p(win-stay), (6) p(lose-shift), (7) relative response time (*shift* vs *stay*), (8) averaged response time, (9) performance over chance level.

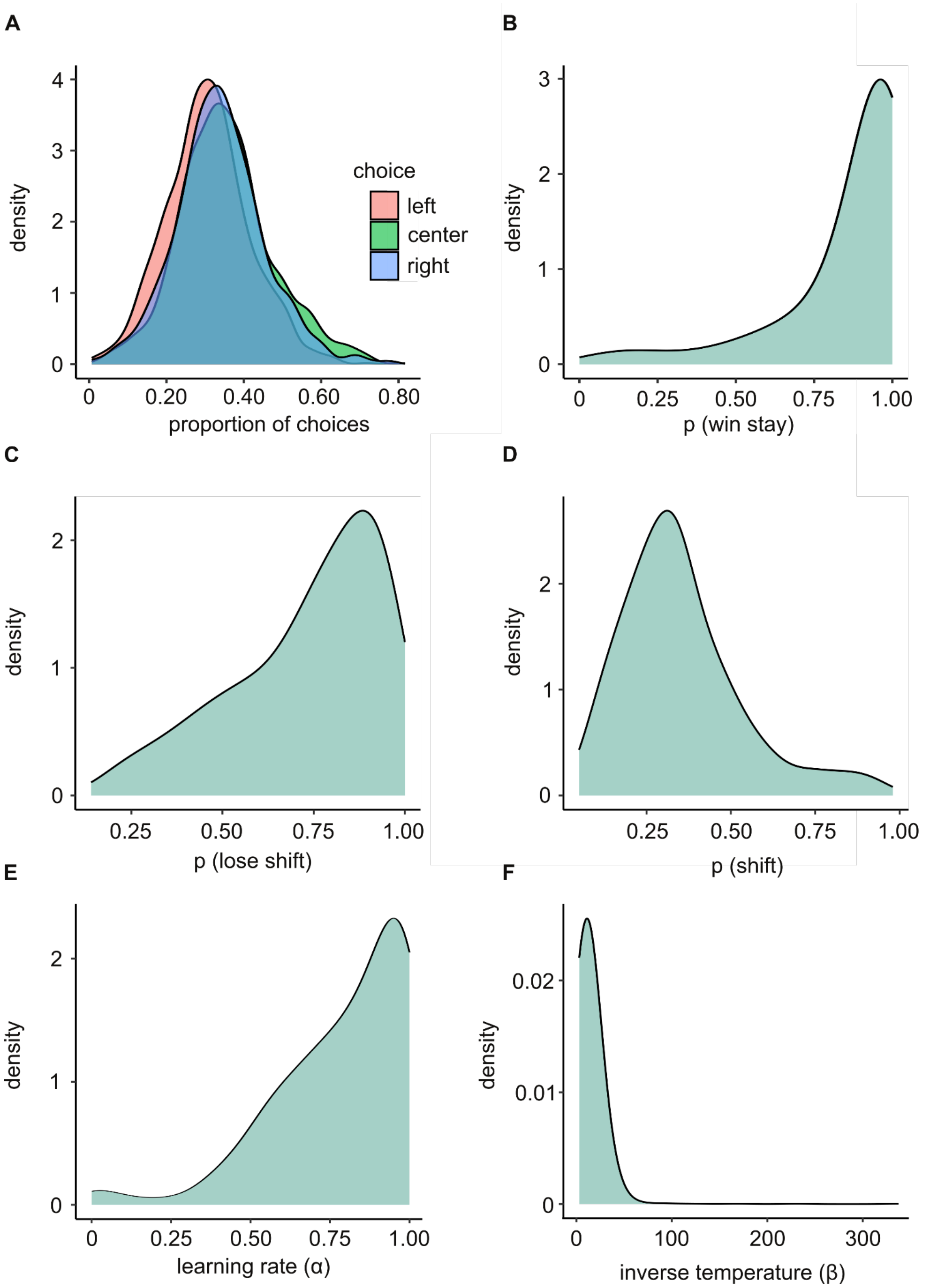

Figure S2. Distributions of choice behavior and RL Parameters.
A) Density plot showing the distribution of choice proportions across the three options (left, center, right). **B)** Distribution of win-stay probability, or the likelihood of repeating a choice following a rewarded trial. **C)** Distribution of lose-shift probability, or the likelihood of switching choices following an unrewarded trial. **D)** Overall shift probability, representing the likelihood of changing choices on any given trial. **E)** Distribution of the learning rate (α), which determines how strongly past outcomes influence future

choices. **F)** Distribution of the inverse temperature (β), which governs the degree of exploitation versus exploration in decision-making. N=1001 for all analyses.

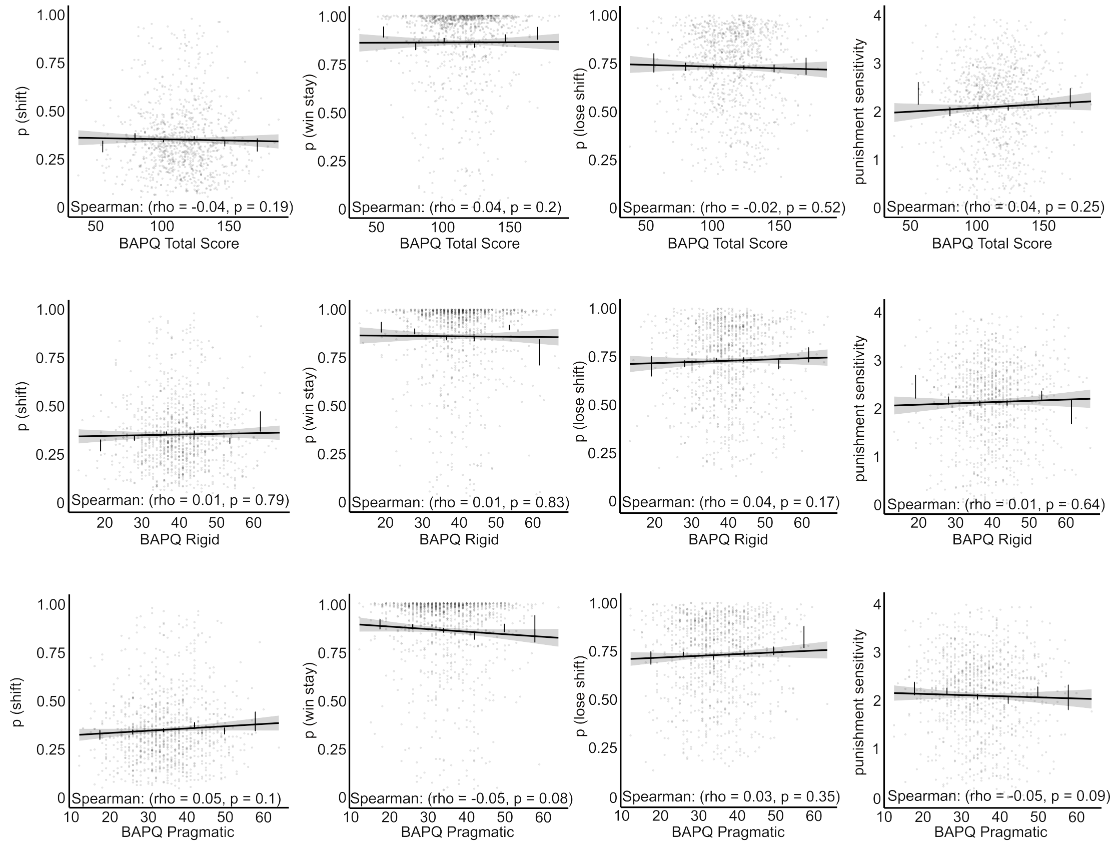

Figure S3. BAPQ Total, Shift, and Pragmatic Scores Do Not Reflect Choice Behavior

A) [Column] Probability of shifting on a given trial relative to BAPQ total, rigid, and pragmatic scores. **B)** [Column] Probability of repeating a choice following reward relative to relative to BAPQ total, rigid, and pragmatic scores. **C)** [Column] Probability of shifting from previous choice following an unrewarded choice relative to BAPQ total, rigid, and pragmatic scores. **D)** [Column] Sensitivity to unrewarded trials relative to rewarded trials, normalized to overall shiftiness of the participant, relative to BAPQ total, rigid, and pragmatic scores. Error bars represent SEM for each bin, shaded areas represent 95% confidence interval for the regression estimate, n=1001 for all analyses.
